# Strigolactone Analogs Are Promising Antiviral Agents for the Treatment of Human Cytomegalovirus Infection

**DOI:** 10.3390/microorganisms8050703

**Published:** 2020-05-10

**Authors:** Matteo Biolatti, Marco Blangetti, Giulia D’Arrigo, Francesca Spyrakis, Paola Cappello, Camilla Albano, Paolo Ravanini, Santo Landolfo, Marco De Andrea, Cristina Prandi, Valentina Dell’Oste

**Affiliations:** 1 Department of Public Health and Pediatric Sciences, University of Turin, 10126 Turin, Italy; matteo.biolatti@unito.it (M.B.); camilla.albano@unito.it (C.A.); santo.landolfo@unito.it (S.L.); 2 Department of Chemistry, University of Turin, 10125 Turin, Italy; marco.blangetti@unito.it (M.B.); cristina.prandi@unito.it (C.P.); 3 Department of Molecular Biotechnology and Health Sciences, University of Turin, 10126 Turin, Italy; giulia.darrigo@unito.it (G.D.); francesca.spyrakis@unito.it (F.S.); 4 Department of Drug Science and Technology, University of Turin, 10125 Turin, Italy; paola.cappello@unito.it; 5 Laboratory Medicine Department, Laboratory of Molecular Virology, “Maggiore della Carità” Hospital, 28100 Novara, Italy; paolo.ravanini@gmail.com; 6 Center for Translational Research on Autoimmune and Allergic Disease-CAAD, 28100 Novara, Italy

**Keywords:** human cytomegalovirus, strigolactone analogs, antiviral activity, apoptosis

## Abstract

The human cytomegalovirus (HCMV) is a widespread pathogen and is associated with severe diseases in immunocompromised individuals. Moreover, HCMV infection is the most frequent cause of congenital malformation in developed countries. Although nucleoside analogs have been successfully employed against HCMV, their use is hampered by the occurrence of serious side effects. There is thus an urgent clinical need for less toxic, but highly effective, antiviral drugs. Strigolactones (SLs) are a novel class of plant hormones with a multifaceted activity. While their role in plant-related fields has been extensively explored, their effects on human cells and their potential applications in medicine are far from being fully exploited. In particular, their antiviral activity has never been investigated. In the present study, a panel of SL analogs has been assessed for antiviral activity against HCMV. We demonstrate that TH-EGO and EDOT-EGO significantly inhibit HCMV replication in vitro, impairing late protein expression. Moreover, we show that the SL-dependent induction of apoptosis in HCMV-infected cells is a contributing mechanism to SL antiviral properties. Overall, our results indicate that SLs may be a promising alternative to nucleoside analogs for the treatment of HCMV infections.

## 1. Introduction

Human cytomegalovirus (HCMV), which is part of the *Betaherpesvirinae* subfamily, is one of the most significant opportunistic human pathogens. Although HCMV rarely causes symptomatic clinical manifestations in immunocompetent individuals, it induces severe morbidity and mortality in the immunocompromised population, following either primary infection or reactivation, leading to gastro-intestinal diseases, pneumonia, retinitis and other organ infections [1]. Moreover, HCMV is the most common cause of congenital malformations in developed countries, resulting in neurodevelopmental delay, fetal and neonatal death, and most frequently sensorineural hearing loss [2,3].

Clinically available drugs for anti-HCMV therapy are currently mainly composed of nucleoside, nucleotide and non-nucleotide inhibitors of viral DNA synthesis [4]. However, these agents suffer from several drawbacks, including the induction of adverse side effects, especially in the treatment of congenital infections, and the selection of single- or multi-resistant HCMV mutants [2]. Therefore, there is a burning need to develop new compounds against HCMV diseases.

Strigolactones (SLs) are a newly emerged class of plant hormones with many functions. They are made up of a tricyclic ABC core bound to a fourth butenolide ring, commonly known as the D-ring, that is generally thought to be responsible for the bioactivity of SLs [5]. SLs contribute to defining plant morphology, even in response to environmental conditions, and are involved in the setup of communication with organisms in the rhizosphere. For instance, they regulate shoot branching and serve as rhizosphere signals for the control of host-plant interactions with heterologous organisms, including symbiotic arbuscular mycorrhizal fungi and parasitic weeds [6]. In recent years, SLs have become a cutting-edge topic in plant biology and agronomy as they hold great potential for the development of modern agriculture [7].

While their role in plant-related fields has been thoroughly investigated, the effects of SLs on human cells and their use in medicine are both still poorly defined. The most significant data reported thus far refer to the effect of SLs on cancer cells [8,9,10]. Indeed, it has been demonstrated that synthetic analogs of SLs induce G2/M arrest and apoptosis in a variety of human cancer cells, while having minimal influence on the growth and viability of non-transformed cells, such as human fibroblasts, mammary epithelial cells and normal primary prostate cells [11,12]. Interestingly, cancer cells with stem-like properties are more sensitive to the inhibitory effects of SL analogs than the heterogeneous population of cancer cells [11]. The SL anti-proliferative effects displayed on cancer cells have also been confirmed by the finding that SLs induce DNA double-strand breaks (DSBs), and impair cellular DNA-repair [13].

Finally, recent papers have reported the promising anti-inflammatory effects that SLs exert by inhibiting the release of inflammatory molecules, i.e., nitric oxide (NO), tumor necrosis factor-alpha (TNF-α) and interleukin-6 (IL-6), and the migration of neutrophils and macrophages in fluorescent-protein-labeled zebrafish larvae [14], as well as by triggering the expression of detoxifying enzymes, such as heme-oxygenase (HO-1) and NAD(P)H dehydrogenase [quinone] 1 (NQO1) [15].

As the antiviral activity of SLs has never been investigated, we have screened a panel of SL analogs in order to identify new druggable targets for anti-HCMV therapy. We show, for the first time, that the SLs TH-EGO and EDOT-EGO and their derivatives that lack the butenolide ring (TH-ABC and EDOT-ABC) (see Table 1) markedly inhibit the replication of different HCMV strains in vitro. Moreover, we demonstrate that SLs do not affect the first steps of HCMV infection, i.e., attachment and entry, rather, they exert their role on the late phases of the viral cycle. In particular, we show that an SL-dependent apoptotic trigger may be a novel strategy against HCMV infection.

Finally, *in silico* molecular docking simulations have been used to predict the interactions between the SL analogs and the modeled structure of the putative target IE1, which is known to inhibit apoptosis [16,17,18].

## 2. Materials and Methods

### 2.1. Compounds

The SL analogs TH-EGO, EDOT-EGO and EGO-10 were synthesized as previously described [19]. TH-ABC and EDOT-ABC were synthesized according to the cited procedure, with the exception that the last synthetic sequence step was omitted. GR24 was purchased from Strigolab srl. 

The SL analogs were dissolved in dimethyl sulfoxide (DMSO) (Sigma-Aldrich, Milan, Italy) at stock concentrations of 20 mM and used as racemic mixtures. Cells were treated at the indicated doses by diluting the compounds to the required highest concentration in the appropriate culture medium.

### 2.2. Cells and Viruses

Primary human foreskin fibroblasts (HFFs, ATCC SCRC-1041™, American Type Culture Collection, Manassas, VA, USA) and African green monkey kidney cells (VEROs, ATCC CCL-81™) were cultured in Dulbecco’s Modified Eagle’s Medium supplemented with 10% heat inactivated fetal bovine serum (FBS), 2 mM glutamine, 1 mM sodium pyruvate, 100 U/mL penicillin and 100 µg/mL streptomycin sulfate (Sigma-Aldrich, Milan, Italy). The HCMV laboratory strain Merlin was kindly provided by Klaus Hamprecht and Gerhard Jahn (University Hospital of Tübingen, Germany) [20]; the VR1814 isolate was provided by Giuseppe Gerna (University of Pavia, Italy) [21]; the AD169 (ATCC VR538, American Type Culture Collection, Manassas, VA, USA) HCMV strain was already available at the Laboratory of Pathogenesis of Viral Infections (University of Turin, Italy). All HCMV strains were propagated and titrated on HFFs using a standard plaque assay [22]. Clinical isolates of HSV-1 and HSV-2 were a generous gift from Valeria Ghisetti, “Amedeo di Savoia” Hospital, Turin, Italy. They were propagated and titrated by plaque assay on Vero cells, as previously described [23].

### 2.3. Antiviral Assays

#### 2.3.1. Virus Yield Reduction Assay

HFF or VERO cells were seeded in 24-well plates and pre-treated, after 24 h, with different concentrations of SL analogs or the vehicle control (DMSO) for 2 h at 37 °C. They were then infected with HCMV or HSV, respectively, at MOI of 0.1 PFU/cell in the presence of SLs. Following virus adsorption (2 h at 37 °C), the viral inoculum was removed, and the cultures were maintained in medium that contained the corresponding molecule for 144 h (HCMV) or 48 h (HSV). The cells and supernatants were then harvested and disrupted using three freeze (liquid nitrogen)/thaw (37 °C) cycles. The extent of virus replication was subsequently assessed by titrating the infectivity of the supernatants of the cell suspensions in HFF or VERO cells, as previously described [23,24]. Plaques were microscopically counted, and the mean plaque counts for each drug concentration were expressed as a percentage of the mean plaque count of DMSO. The number of plaques was plotted as a function of drug concentration, and the concentration that produced a 50% reduction in plaque formation (IC_50_) was determined for each test by a nonlinear regression (curve fitting analysis) in GraphPad Prism software. 

#### 2.3.2. Attachment Assay

HFFs were seeded in 24-well plates and, after 24 h, the cultures were chilled over ice for 20 min and washed three times with a 4 °C pre-chilled medium. Pre-chilled cell monolayers were then treated with either serial dilutions of compounds or DMSO for 30 min at 4 °C, then infected with on ice pre-cooled HCMV (MOI 0.1) and incubated for 2 h at 4 °C to ensure viral attachment, but not entry. After two gentle washes with cold DMEM to remove any unattached virus and compounds, cells were overlaid with 0.8% methylcellulose medium and shifted to 37 °C for 72 h. At the end of the incubation, plates were fixed and colored with crystal violet, and the plaques were microscopically counted. Subsequently, one lane per plate was used as a control to confirm that incubation at 4 °C allowed viral attachment, but not viral entry. In order to establish this, the cells to which the virus had been pre-attached at 4 °C were treated with cold (4 °C) acidic glycine (100 mM glycine, 150 mM NaCl [pH 3]) for 2 min to inactivate any attached, but not yet penetrated, virus, before being overlaid with 0.8% methylcellulose. This resulted in 100% inhibition of plaque formation in untreated cells, indicating that no virus had entered the cells during the attachment period.

#### 2.3.3. Entry Assay

HCMV (MOI of 0.1) was adsorbed for 2 h at 4 °C on pre-chilled (4 °C) confluent HFFs to allow viral attachment. Cells were then washed with cold DMEM three times to remove any unbound virus, treated with different concentrations of either the compounds or DMSO, and incubated for 2 h at 37 °C. Any un-penetrated virus was inactivated with acidic glycine for 2 min at room temperature, as previously described. Subsequently, the cells were washed three times with medium pre-warmed at 37 °C to return the pH to neutral, overlaid with 0.8% methylcellulose and incubated for 72 h at 37 °C. Plates were then fixed and colored with crystal violet, and plaques were microscopically counted. 

### 2.4. Cytotoxicity Assay

To determine SL cytotoxicity, HFF and VERO cells were seeded in a 96-well culture plate and exposed to increasing concentrations of either SLs or vehicle (DMSO) the following day. After 144 h or 48 h, respectively, of incubation, the number of viable cells was determined using the 3-(4,5-dimethylthiazol-2-yl)-2,5-diphenyltetrazolium bromide (MTT) (Sigma-Aldrich, Milan, Italy) assay, as previously described [15].

### 2.5. Western Blot Analysis

After treatment, cells were washed with PBS, and cell lysis was carried out using Radioimmunoprecipitation Assay (RIPA) buffer to obtain total cell lysate. An equal amount of the cell extracts was fractionated by electrophoresis on sodium dodecyl sulfate polyacrylamide gels and transferred to Immobilon-P membranes (Biorad, Milan, Italy). After blocking with 5% nonfat dry milk in TBS-Tween 0.05%, membranes were incubated overnight at 4 °C with the appropriate primary antibodies. The following primary antibodies were used: mouse monoclonal antibodies anti-IEA (IE1 and IE2, clone CH160) (Virusys, Taneytown, MD, USA, P1215), UL44 (clone (CH13) (Virusys, Taneytown, MD, USA, P1202-1), pp28 (clone 5C3) (Virusys, Taneytown, MD, USA, CA004-100) and anti-α-Tubulin (clone 5-B-1-2) (Active-Motif, La Hulpe, Belgium, 39527) (all at 1:1000 dilution in 5% nonfat dry milk, TBS-Tween 0.05%). After washing with TBST buffer (500 mM NaCl, 20 mM Tris pH 7.4, 0.05% Tween 20), the membrane was incubated with an HRP-conjugated anti-mouse secondary antibody (GE Healthcare, Chicago, IL, USA) for 1 h at room temperature, and visualized using an enhanced chemiluminescence detection kit (SuperSignal West Pico Chemiluminescent Substrate, Thermo SCIENTIFIC, Waltham, MA, USA).

### 2.6. Apoptosis Detection

#### 2.6.1. Annexin V Analysis

To distinguish apoptotic from necrotic cells, double staining was performed for exposed phosphatidylserine and propidium iodide (PI) exclusion using the Annexin V-FITC Apoptosis Detection Kit (Calbiochem, San Diego, CA, USA). Experiments were performed according to the manufacturer’s instructions. Briefly, the different compounds (12.5 µM) were added to the HFFs, and the cells were then infected and processed 24 or 48 h after treatment. The cells were washed in PBS, trypsinized and then resuspended in a binding buffer (10 mM HEPES/NaOH, pH 7.4, 140 mM NaCl, 2.5 mM CaCl_2_). Annexin V-FITC was added to a final concentration of 100 ng/mL, and the cells were incubated in the dark for 10 min, then washed again in PBS and resuspended in 300 μL of the binding buffer. In total, 40 μg/mL of PI was added to each sample before the flow cytometric analyses. Cells were analyzed using a FACSCalibur flow cytometer (BD Biosciences, Franklin Lakes, NJ, USA). Data analysis was performed using standard ModFit LT software (BD Biosciences, Franklin Lakes, NJ, USA). Unstained cells, cells stained with Annexin V-FITC only and cells stained with PI only were used as controls to establish compensation and quadrants. Cells were gated according to their light-scatter properties to exclude cell debris. 

#### 2.6.2. In-Vitro Analysis of Caspase-3 Activity

Caspase-3 activity was assessed using the SensoLyte AFC Caspase Sampler Kit “Fluorimetric” (Anaspec, CA, USA). Experiments were performed according to the manufacturer’s instructions. After 1 h of incubation at 25 °C, fluorescence was measured at an excitation wavelength of 405 nm and an emission wavelength of 500 nm using the VICTOR3 1420 multilabel counter (Perkin–Elmer, Milan, Italy). Protease activity was expressed as RFU (relative units of fluorescence).

### 2.7. Molecular Modeling

The HCMV IE1 (UL123) structure was modeled by means of the homology modeling service SWISS-MODEL [25]. The crystal structure of the IE1 of the rhesus macaque cytomegalovirus (PDB code 4wic) was used as a template to construct the model of the IE1 of HCMV (Merlin strain). The identity sequence was 25% and the coverage 74%. The stereochemical validation of the model was performed by building the Ramachandran plot of the protein-backbone using the RAMPAGE server (http://mordred.bioc.cam.ac.uk/~rapper/rampage.php). Possible druggable pockets were identified in the protein model using the FLAP*site* algorithm, which was implemented in the FLAP software (Molecular Discovery Ltd., Borehamwood, UK) [26]. The molecular docking of SL analogs was performed using GOLD suite version 5.5 (The Cambridge Crystallographic Data Centre, CCDC, Software Ltd., Cambridge, UK) [27]. The region of interest was defined within 10 Å from a reference atom (HB2 on Arg72). GOLD default parameters were set, and the compounds were subjected to 15 genetic algorithm runs using the CHEMPLP fitness function. Pictures were prepared using PyMOL version 1.7.6.4.

### 2.8. Statistical Analysis

All statistical tests were performed using GraphPad Prism version 5.00 for Windows (GraphPad Software, La Jolla, CA, USA). Data are presented as means ± standard deviations (SD). Means were compared using two-way analysis of variance (ANOVA) with Bonferroni’s post-tests. Differences were considered to be statistically significant for *p* < 0.05 (*, *p* < 0.05; **, *p* < 0.01; ***, *p* < 0.001).

## 3. Results

### 3.1. Effects of SL Analogs on HCMV Productive Infection

In order to identify novel compounds that are capable of inhibiting HCMV replication in vitro, we screened a series of SL analogs, named TH-EGO, EDOT-EGO, EGO-10 and GR24, which had previously been characterized for their anti-proliferative [11,12] and anti-inflammatory activities [13,14,15]. Their chemical characteristics are reported in Table 1.

Although the stereochemistry of SLs at the 2′ position of the butenolide ring and the fact that it plays a crucial role in bioactivity effectiveness have been well explored, we decided to start our preliminary screening with racemic mixtures of the compounds [28].

**Figure 1:**
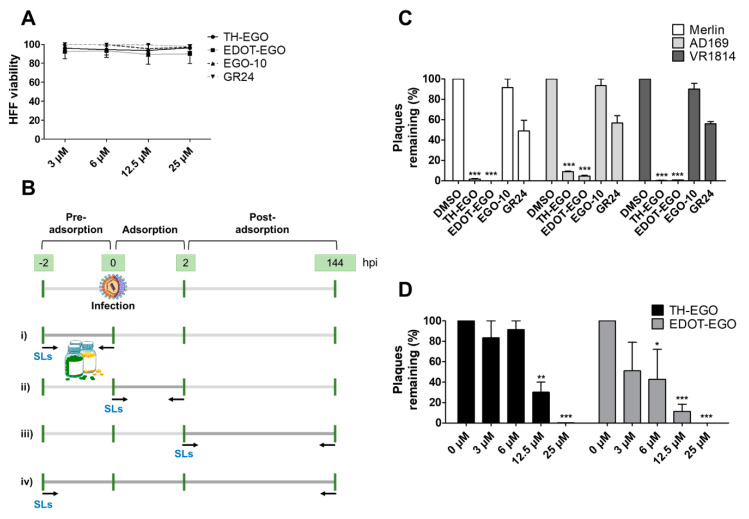
SL analogs antiviral activity against the human cytomegalovirus (HCMV). (**A**) Cell viability was measured using the 3-(4,5-dimethylthiazol-2-yl)-2,5-diphenyltetrazolium bromide (MTT) assay. Confluent human foreskin fibroblast (HFF) cultures seeded in 96-well plates were incubated with different concentrations of the indicated SLs for 144 h. Graphs are representative of three independent experiments with duplicate replicate wells for each analysis. (**B**) Flow-charts of time-of-addition assays. (**C**) Identification of SL-analogs with anti-HCMV activity. HFF cells were cultivated in 24-well plates and pre-treated with 25 µM of the indicated SLs for 2 h. Subsequently, cells were infected with the indicated HCMV strains (MOI of 0.1) and, following virus adsorption (2 h at 37 °C), the viral inoculum was removed; cultures were exposed to SLs during the infection, for 144 h. Dimethyl sulfoxide (DMSO) was used as the vehicle control. The extent of HCMV replication was then assessed by titrating the infectivity of supernatants and cell-associated viruses—obtained from freeze (liquid nitrogen)/thaw (37 °C) cycles—and combined using a standard plaque assay in HFFs. Plaques were microscopically counted, and the mean plaque counts for each molecule were expressed as a percentage of the mean plaque count of the control. Three independent experiments were performed, and one representative experiment is shown (***, *p* < 0.001, two-way ANOVA followed by Bonferroni’s post-tests). (**D**) HFFs were infected with HCMV (Merlin strain, MOI of 0.1) and, where indicated, the cells were treated with increasing concentrations of TH-EGO, EDOT-EGO or DMSO, before and during virus adsorption. These remained in the culture media throughout the experiment. The extent of HCMV replication was then assessed by titrating the infectivity of supernatants and cell-associated viruses—obtained from freeze (liquid nitrogen)/thaw (37 °C) cycles—and combined using a standard plaque assay. Plaques were microscopically counted, and the mean plaque counts for each drug concentration were expressed as a percentage of the mean count of the control. The number of plaques was plotted as a function of drug concentration. Three independent experiments were performed, and one representative experiment is shown (*, *p* < 0.05; **, *p* < 0.01; ***, *p* < 0.001, two-way ANOVA followed by Bonferroni’s post-tests).

Prior to the assessment of the antiviral activity of SL analogs, a standard MTT viability assay was performed to rule out the possibility that the drugs may have cytotoxic effects on the HFF cells. To this end, different concentrations of each molecule were tested, and the compounds were only considered non-toxic if they maintained at least 70% cell viability after 144 h of treatment. This parameter indicated that the cytotoxicity of the compounds shown in Figure 1A (i.e., TH-EGO, EDOT-EGO, EGO-10 and GR24) for primary HFFs was low or undetectable at concentrations of up to 25 µM as ~90% of treated cells were viable after 144 h (Figure 1A).

TH-EGO, EDOT-EGO, EGO-10 and GR24 were then analyzed for their antiviral activity against a range of HCMV strains, i.e., Merlin, AD169 and VR1814, at a concentration of 25 µM, which was the minimum effective dose that did not show particular cytotoxicity. A series of time-of-addition assays were performed to show which phase of the HCMV replication cycle is targeted by SL analogs. Briefly, the compounds were added to the cells as follows: (i) before infection (pre-adsorption stage, from 2 h prior to infection); (ii) during infection (adsorption stage, 2 h); (iii) after infection (post-adsorption stage, from 0 to 144 hpi); or (iv) before, during, and after infection (Figure 1B). The virus yield reduction assay revealed that TH-EGO, EDOT-EGO and to a lesser extent GR24 demonstrated significant activity against HCMV when added before, during and after infection (Figure 1C). Finally, the inhibition of HCMV replication was limited to the higher doses tested or was absent in the pre-treatment or post-treatment assays, meaning that IC_50_ values could not be determined (data not shown). Two of the SL analyzed, namely TH-EGO and EDOT-EGO, inhibited HCMV replication by over 90% and were, thus, selected for further analysis. Interestingly, the inhibitory activity of SLs was not strain specific, as was observed in HFFs infected with different HCMV strains (Figure 1C).

A more detailed analysis with serial dilutions of the two compounds (3–25 μM) revealed that TH-EGO and EDOT-EGO specifically inhibited HCMV replication (the Merlin strain was used in all of the subsequent experiments, because its background resembles clinical isolates [29]) in a dose-dependent manner, with inhibitory concentration (IC_50_) values of 12.04 and 9.13 μM, respectively (Figure 1D).

To further evaluate whether the antiviral activity of the SL analogs was limited to HCMV or whether it could be extended to other members of the *Herpesviridae* family, we assessed their efficacy against herpes simplex type 1 (HSV-1) and type 2 (HSV-2) (Figure S1). MTT viability assays on VERO cells after 48 h of treatment were used to establish that a dose of 12.5 µM was associated with low or undetectable cytotoxicity for all of the compounds tested (Figure S1, panel A). We confirmed that TH-EGO and EDOT-EGO were the most powerful SL analogs against both HSV-1 and HSV-2, with IC_50_ values of 5.71 and 11.80 for TH-EGO, and 4.16 and 3.81 for EDOT-EGO, respectively (Figure S1, panel B and C).

### 3.2. The Butenolide Ring Is Critical for the Antiviral Activity of TH-EGO and EDOT-EGO

**Figure 2:**
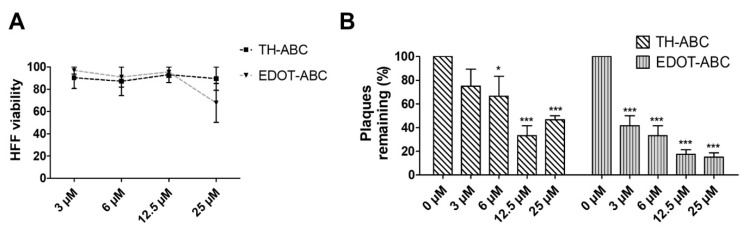
SL derivatives antiviral activity against HCMV. (**A**) Viability was measured using the MTT assay. Confluent HFF cultures were seeded in 96-well plates and incubated with different concentrations of SLs for 144 h. Graphs are representative of three independent experiments with duplicate replicate wells for each analysis. (**B**) Antiviral activity of TH-ABC and EDOT-ABC on HCMV replication. HFF cells were pre-treated with the indicated dilutions of SLs for 2 h. Subsequently, the HFFs were infected with the HCMV Merlin strain (MOI of 0.1) and, following virus adsorption (2 h at 37 °C), the viral inoculum was removed and the cultures were exposed to increasing dilutions of either the SLs or vehicle (DMSO) and incubated for 144 h. Supernatants and cell-associated viruses—obtained from freeze (liquid nitrogen)/thaw (37 °C) cycles—were combined, and virus infectivity titers were determined by plaque assay. Plaques were microscopically counted, and the mean plaque counts for each compound concentration were expressed as a percentage of the mean count of the control (*, *p* < 0.05; ***, *p* < 0.001, two-way ANOVA followed by Bonferroni’s post-tests).

Since TH-EGO and EDOT-EGO displayed the most potent anti-HCMV activity, we sought to unveil the functional group responsible for this antiviral property. Two SL analogs that lack an enol ether bridge on the butenolide ring, named TH-ABC and EDOT-ABC, were then synthesized and evaluated for their antiviral activity (Table 1). As shown in Figure 2A, the modified compounds also did not affect (TH-ABC, ~90%) or slightly affect (EDOT-ABC, ~70%) cell viability up to 25 µM. Interestingly, they partially retained antiviral activity against HCMV in vitro, although it was lower than that of the original compounds, suggesting that the presence of an enol ether bridge on the butenolide ring is critical for the antiviral activity of TH-EGO and EDOT-EGO (Figure 2B).

Interestingly, the efficacy of the SL derivatives that lack the enol ether bridge on the butenolide ring (TH-ABC and EDOT-ABC) were highly compromised for both HSV-1 and HSV-2, confirming that this structure is critical to the antiviral activity of TH-EGO and EDOT-EGO (Figure S1, panel C).

### 3.3. TH-EGO and EDOT-EGO Inhibit an Early-Late Event in the HCMV Replication Cycle

**Figure 3:**
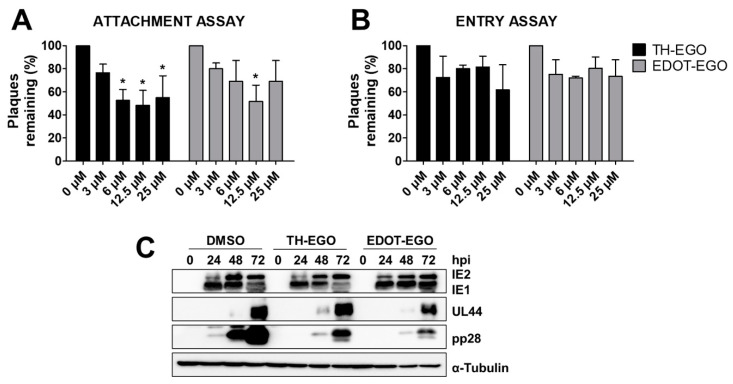
Inhibitory effect on individual stages of viral protein production. (**A**) Attachment assay. HFF cells were infected with HCMV Merlin (MOI of 0.1) in the presence of serial dilutions of either the SLs or vehicle (DMSO); inoculated cultures were then kept for 2 h at 4 °C in presence of SL analogs to allow virus attachment, but not entry, to occur, which were then tested using plaque reduction assays. Plaques were microscopically counted, and the mean plaque counts for each drug concentration were expressed as a percentage of the mean count of the control (*, *p* < 0.05, two-way ANOVA followed by Bonferroni’s post-tests) (**B**) Entry assay. HFFs were infected in the absence of SLs and kept at 4 °C for 2 h to allow virus attachment to occur; serial dilutions of the compound were added to washed cells and the temperature was then shifted to 37 °C to allow entry to occur. After a single wash with acidic glycine to remove virus particles from the cell surface, cells were overlaid with methylcellulose-containing medium. Data are presented as a percentage of the mean count of the control (two-way ANOVA followed by Bonferroni’s post-tests). (**C**) HFFs were treated with TH-EGO, EDOT-EGO (12.5 µM) or with equal volumes of DMSO solvent 1 h before infection and for the entire duration of the infection, and infected with HCMV at a MOI of 0.3. Lysates were prepared at the indicated time-points and subjected to Western blot analyses for IEA (IE1 + IE2), UL44, pp28 and α-Tubulin, which was used as a loading control.

In order to gain more insight into the nature of the antiviral activity of the selected SL analogs, the effects of TH-EGO and EDOT-EGO were investigated during the HCMV replication cycle. To investigate if the compounds may target the early steps of the virus life cycle, i.e., virus attachment and entry into cells, an attachment assay was first carried out under experimental conditions in which the virus was allowed to bind to the surface of the host cells, in the presence or absence of SLs, but did not undergo cell entry. Briefly, pre-chilled HFF monolayers were infected with HCMV in the presence of the SLs, for 2 h at 4 °C. Cells were washed to remove the compounds and any unabsorbed viral particles, and a 0.8% methylcellulose solution was then added to measure the infectivity of the particles that had successfully attached to the cells. As shown in Figure 3A, TH-EGO and EDOT-EGO barely impaired the attachment of HCMV in a concentration-independent manner.

To test whether the inhibitory activities of the SL analogs were due to the inhibition of HCMV entry into cells, pre-chilled HFF monolayers were infected with HCMV for 2 h at 4 °C. TH-EGO, EDOT-EGO and DMSO (used as a vehicle negative control) were then added, and the cells were further incubated at 37 °C to allow viral entry. These experimental conditions allow synchronized virus penetration to occur following attachment at low temperatures. After 2 h at 37 °C, SL analogs were removed, and acidic glycine treatment was performed to inactivate any residual HCMV particles that were attached to the cell surface. The cells were then overlaid with 0.8% methylcellulose to measure the infectivity of HCMV that had successfully entered the cells. As shown in Figure 3B, SL analogs only weakly affected HCMV entry under the examined concentrations. Taken together, these findings suggest that TH-EGO and EDOT-EGO are not able to interfere with viral attachment and entry, indicating that the initial steps of HCMV replication are not the main targets for SLs.

Finally, in order to identify the phase of the HCMV replication program that is affected by SL analogs, the effects of TH-EGO and EDOT-EGO on viral gene expression were investigated in HCMV-infected cells. To this end, total protein cell extracts were prepared from HCMV-infected HFFs that had been treated with the indicated compounds for various lengths of time post infection. The expression patterns of IEA (IE1 and IE2), UL44 and pp28 were then examined via immunoblotting with specific antibodies and were used as a reflection of the levels of immediate-early, early and late viral products, respectively. As shown in Figure 3C, TH-EGO and EDOT-EGO decreased the expression of the late tegument protein pp28 at every time point, whereas the IEA and UL44 proteins were not affected. These results indicate that the SL analogs interfere with a molecular event that occurs in an early-late stage of the HCMV replication cycle, i.e., a stage that follows the onset of IE gene expression and viral DNA replication.

### 3.4. Virolysis as the Mechanism for TH-EGO and EDOT-EGO Antiviral Activity

**Figure 4:**
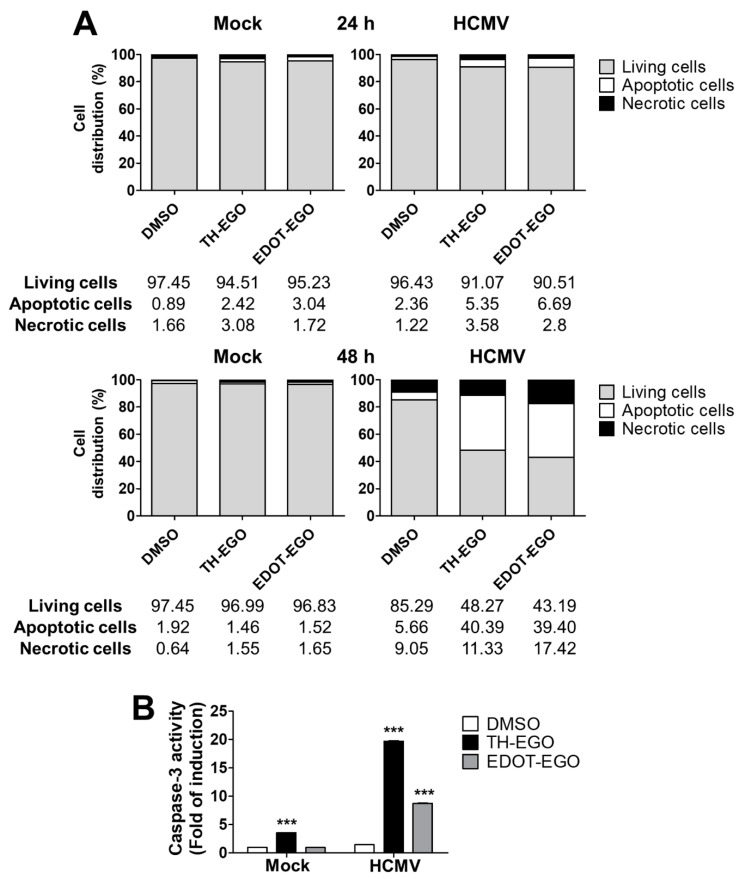
Interplay between apoptosis and SL analogs. (**A**) HFFs were treated with TH-EGO, EDOT-EGO (12.5 µM) or with equal volumes of DMSO solvent 1 h before infection and for the entire duration of the infection, and infected with HCMV (Merlin strain, MOI 1). After 24 h (upper panel) and 48 h (lower panel), cells were processed for Annexin V/propidium iodide (PI) flow cytometric analysis. Annexin−/PI− cells indicated living cells, Annexin+/PI− apoptotic cells and Annexin+/PI+ necrotic cells. Values were plotted as the percentage of cell distribution across the three different conditions. (**B**) HFFs were treated as described in (**A**) for 24 h with either SL TH-EGO, EDOT-EGO (12.5 µM) or with the solvent DMSO, in the absence or the presence of HCMV (Merlin strain, MOI 1) and processed via fluorimetric assay for caspase-3 activation. Fluorescence intensity is reported as RFU values (relative fluorescence units). Fold changes were calculated after the normalization of the SL-analogs vs. DMSO-treated cells, in mock or HCMV-infected cells. Data are shown as mean ± SD (***, *p* < 0.001, two-way ANOVA followed by Bonferroni’s post-tests).

It has been previously reported that SL analogs induce apoptosis in the osteosarcoma cell line U2OS [13]. As apoptosis is also one of the major mechanisms by which hosts evade viral infection [18,30], we assessed the capability of SL analogs to modulate cell-death pathways in HCMV-infected cells via dual staining with annexin V and PI. The binding of Annexin V to phosphatidyl serine is a marker of early apoptosis, while PI only stains the chromatin of cells with compromised plasma membrane and is, therefore, a marker of necrosis. As shown in Figure 4A, at 48 hpi, a significant increase in early apoptosis (annexin+/PI−) and necrosis (annexin+/PI+) was detected in cells treated with TH-EGO (40.39 vs. 5.66; 11.33 vs. 9.05) and EDOT-EGO (39.40 vs. 5.66; 17.42 vs. 9.05) compared to vehicle-treated cells. Moreover, we compared the activity of caspase-3, the final executor of the apoptotic cascade, in cells treated with either TH-EGO, EDOT-EGO or DMSO at different time points after HCMV infection to definitively corroborate the pro-apoptotic effect of SLs on infected cells, as caspases play a central role in mediating various apoptotic responses. Interestingly, significant differences in the ability to activate caspase-3 were observed in SL-treated, infected and non-infected cells (Figure 4B), suggesting that the SLs may specifically trigger caspase activity and promote the apoptotic process only during the course of HCMV infection.

### 3.5. Identification of Putative Drug-Binding Target Proteins Using In-Silico Docking Simulations

**Figure 5:**
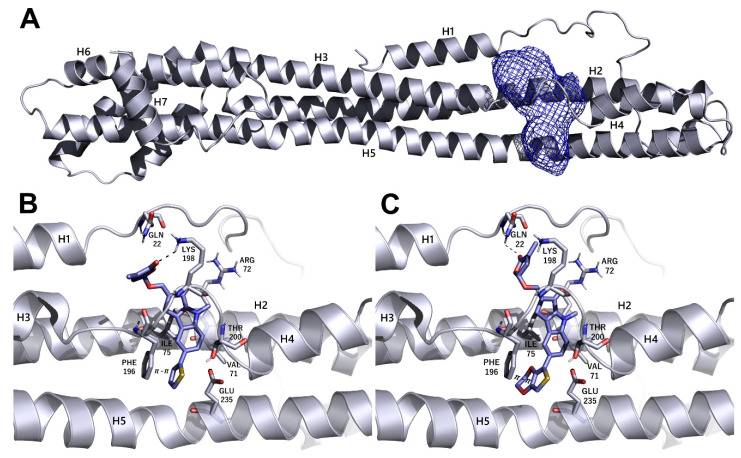
Modeling of the possible complexes formed by IE1 and the most active SL analogs TH-EGO and EDOT-EGO. (**A**) Homology model of IE1. The protein is represented as a cartoon and the selected binding site as a mesh contour. (**B**,**C**) Predicted binding pose of TH-EGO and EDOT-EGO in the protein pocket. Hydrogen bonds are shown as black dashed lines. The SLs and the residues lining the pocket are displayed as lilac and grey capped sticks, respectively.

We decided to use an *in silico* structure-based approach to get clues on the possible molecular targets of the SL-analogs, performing molecular docking simulations on possibly relevant HCMV targets. In the series of HCMV proteins involved in apoptosis inhibition, it was found that vMIA (UL37, prevents the pro-apoptotic cascade), vICA (UL36, prevents caspase-8 activation), UL38 (inhibits apoptosis and facilitates viral replication), IE1 (UL123) and IE2 (UL122) play a key role in initiating lytic cycle gene regulation pathways [16]. We searched the Protein Data Bank [31] for the crystallographic structure of the listed proteins and did not find any of them. Therefore, we tried to model the structures of the missing proteins using homology modeling, and were only able to obtain reliable models for IE1 (see Materials and Methods for further details on IE1). The obtained models were analyzed using the FLAP*site* algorithm [26] for the identification of possible binding sites, and the SL analogs were docked in most druggable pockets using GOLD software [32]. Herein, we only report the results that were obtained upon docking the most active SLs, TH-EGO and EDOT-EGO, in IE1. The IE1 structure was modeled on the IE1 of rhesus macaque cytomegalovirus (PDD code 4wic), with which it shares low sequence identity (25%) but high coverage (74%). The model Ramachandran plot reported that 98% of the residues were in favored regions (see Figure S2). The protein model is shown in Figure 5A and displays a relatively elongated structure. The unique pocket, which is large enough to accommodate SL analogs, was identified between helix 2 and the loop connecting helices 3 and 4. TH-EGO and EDOT-EGO were docked in the pocket and their binding pose is reported in Figure 5B,C. Considering that, currently, all the known natural SLs have been shown to have a (*R*)-configuration at the 2′ position where the D-ring is bound to the rest of the molecule [33], in both cases the (*R*)-enantiomer of our synthesized analogues was chosen. The score, which was assigned to the poses using the CHEMPLP scoring function, as implemented in the GOLD software, was quite high ([67 and 70, respectively]; [34]) and supported the reliability of the prediction.

The best poses obtained for the two compounds are quite similar. In the case of TH-EGO, the butenolide ring forms a hydrogen bond with Lys198, while the thiophen moiety is involved in π−π stacking contact with the aromatic ring of Phe196. Hydrophobic interactions are formed between the tricyclic ring ABC and Val71 and Ile75 (Figure 5B). EDOT-EGO hydrogen bonds through the butenolide ring to Gln22. Indeed, the orientation of the butenolide ring is different than that of TH-EGO, and this is the only difference between the poses of the two SL analogs. As for EDOT-EGO, π−π stacking is formed between the ethylenedioxythienyl group and the Phe196 side-chain, and the tricyclic ring ABC forms hydrophobic contact with the same Val71 and Ile75 (Figure 5C). The other SL analogs only maintained the π-π interaction with Phe196, while the hydrogen bonds with the upper part of the binding site are lost. The corresponding CHEMPLP scores were 57 for TH-ABC, 62 for EDOT-ABC, 57 for EGO-10 and 53 for GR24. We believe that the loss of such a polar interaction could be an explanation for the lower activity of the other SLs analogs (Figure S3).

## 4. Discussion

The identification and validation of new antiviral drugs against HCMV replication is a priority for the clinical management of HCMV infections. HCMV is the principal pathogen in transplant recipients and is recognized as the leading viral cause of birth defects [1,2,3]. The standard therapy for HCMV disease is associated with adverse side effects, and prolonged treatment may lead to the emergence of drug-resistant mutants [35]. Moreover, the antivirals that are currently used are not able to prevent the reactivation of latent HCMV, and no vaccine is currently available. Therefore, there is an urgent need for new first-line anti-HCMV drugs with novel mechanisms of action that can be safely administered without causing major adverse effects.

Known for their pleiotropic regulatory roles in plant growth and development, SL analogs are attractive new antiviral candidates that display features that are promising for pharmacology, as shown in the treatment of a variety of solid and non-solid tumors [7].

To the best of our knowledge, this is the first study that demonstrates that SLs have an antiviral effect against various members of the *Herpesviridae* family, including HCMV, HSV-1 and HSV-2. Of the SL analogs analyzed, TH-EGO and EDOT-EGO showed a significant dose-dependent effect upon infection with a range of HCMV strains. Moreover, we have demonstrated herein that their antiviral activity relies on the butenolide ring, as the TH-EGO and EDOT-EGO derivatives that lack this domain displayed lower activity than the unmodified compounds.

A detailed analysis performed over the time course of the infection process has allowed us to demonstrate that the SLs analogs did not affect the very first steps of HCMV replication (attachment and entry). Accordingly, the expression levels of immediate early and early viral proteins were not inhibited by the treatment of HFF with TH-EGO and EDOT-EGO. By contrast, late viral proteins, the paradigm of which is the UL99 gene product (pp28 protein), were significantly affected by the same compounds. Since HCMV replication is a complex process that is regulated by the precisely coordinated interplay of several viral and cellular proteins, our findings support the view that, even if SLs possess inhibitory effects on viral events, the exploitation of other cellular processes may be a suitable strategy with which to identify the cellular pathways targeted by SLs. In this context, further investigations on physiologically important targets for HCMV infection, such as endothelial and epithelial cells, and on cells that do not progress to a lytic infection, such as monocytes, will be crucial to corroborate and expand the data obtained on HFFs.

SL analogs have displayed pro-apoptotic activity in a variety of human cancer cells, but not in non-transformed human fibroblasts [11,12,13]. In this context, the pro-apoptotic activity of SLs relies on their ability to induce the activation of p38 and stress-response pathways [11,12]. Accordingly, we have demonstrated herein that SLs trigger caspase induction and an apoptotic phenotype in HCMV-infected fibroblasts, thus specifically favoring the elimination of infected cells.

During the course of evolution, HCMV has established a complex scenario of viral mechanisms of escape from apoptosis, involving several viral proteins, such as vMIA (UL37 exon 1), vICA (UL36), UL38, IE1 and IE2 [18]. We therefore hypothesized that SL-triggered apoptosis in infected cells relies on specific binding to viral cell-death inhibitors. Molecular docking studies, performed on the homology model of IE1, support the hypothesis that SLs may directly target antiapoptotic proteins when exerting their antiviral activity. The binding poses obtained for TH-EGO and EDOT-EGO in IE1 are consistent, and the estimated energy of interaction is reliable for the formation of a stable protein-ligand complex [34]. However, our findings do not preclude the possibility that SLs may target other antiapoptotic proteins, such as vMIA, cICA, UL38 and IE2, for which no structure, nor even a template to be used for homology modeling, is available. Indeed, structure-based drug design simulations are strongly limited by the availability of a target model. Moreover, other HCMV proteins that we are currently not able to model may be contemporaneously targeted by SLs.

## 5. Conclusions

In summary, our results indicate that TH-EGO and EDOT-EGO are attractive candidates for a new class of antiviral drugs. Antiviral therapy that is based on molecules exerting their effects by targeting cellular proteins instead of specific viral proteins is a promising solution, as they are not associated to drug resistance. The potent SL in vitro antiviral activity warrants further in vivo studies that can validate the potential use of SLs in the prevention and/or control of HCMV infections.

## 6. Patents

Patent “Strigolattoni per uso nella prevenzione e/o trattamento di infezioni da virus della famiglia Herpesviridae” (No: 102018000010142, University of Turin, Italy).

## Figures and Tables

**Table 1:** Strigolactone (SL) compounds used in this study.

Chemical Structure	Compound	IUPAC Name
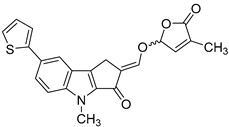	TH-EGO	(±)(*E*)-4-methyl-2-(((4-methyl-5-oxo-2,5-dihydrofuran-2-yl)oxy)methylene)-7-(thiophen-2-yl)-1,4-dihydrocyclopenta[b]indol-3(2*H*)-one
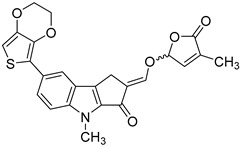	EDOT-EGO	(±)(*E*)-7-(2,3-dihydrothieno[3,4-b][1,4]dioxin-5-yl)-4-methyl-2-(((4-methyl-5-oxo-2,5-dihydrofuran-2-yl)oxy)methylene)-1,4-dihydrocyclopenta[b]indol-3(2*H*)-one
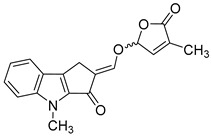	EGO-10	(±)(*E*)-4-methyl-2-(((4-methyl-5-oxo-2,5-dihydrofuran-2-yl)oxy)methylene) -1,4-dihydrocyclopenta[b]indol-3(2*H*)-one
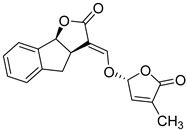	GR24	(±)(3a*R*,8b*S*,*E*)-3-((((*R*)-4-methyl-5-oxo-2,5-dihydrofuran-2-yl)oxy)methylene)-3,3a,4,8b-tetrahydro-2*H*-indeno[1,2-b]furan-2-one
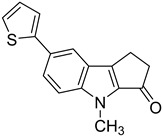	TH-ABC	4-methyl-7-(thiophen-2-yl)-1,4-dihydrocyclopenta[b]indol-3(2*H*)-one
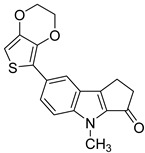	EDOT-ABC	7-(2,3-dihydrothieno[3,4-b][1,4]dioxin-5-yl)-4-methyl-1,4-dihydrocyclopenta[b]indol-3(2*H*)-one

## Supplementary Materials

The following are available online at https://www.mdpi.com/2076-2607/8/5/703/s1.
