# Supplementary material for: Strigolactone Analogs Are Promising Antiviral Agents for the Treatment of Human Cytomegalovirus Infection
Source: Microorganisms. 2020 May 10;8(5):703. doi: 10.3390/microorganisms8050703 (PMC7284764; doi:10.3390/microorganisms8050703)
Supplement: Supplementary file 1 [file microorganisms-08-00703-s001.pdf]

microorganisms

Supplementary Information

Strigolactone Analogs are Promising Antiviral Agents for the Treatment of Human Cytomegalovirus Infection

Matteo Biolatti [1], Marco Blangetti [2], Giulia D'Arrigo [3], Francesca Spyrakis [3], Paola Cappello [4], Camilla Albano [1], Paolo Ravanini [5], Santo Landolfo [1], Marco De Andrea [1,6*], Cristina Prandi [2], and Valentina Dell'Oste [1*].

1 Department of Public Health and Pediatric Sciences, University of Turin, 10126 Turin, Italy; matteo.biolatti@unito.it (M.Bi.); camilla.albano@unito.it (C.A.); santo.landolfo@unito.it (S.L.)
2 Department of Chemistry, University of Turin, 10125, Turin, Italy; marco.blangetti@unito.it (M.Bl.); cristina.prandi@unito.it (C.P.)
3 Department of Molecular Biotechnology and Health Sciences, University of Turin, 10126 Turin, Italy; paola.cappello@unito.it (P.C.)
4 Department of Drug Science and Technology, University of Turin, 10125 Turin, Italy giulia.darrigo@unito.it (G.D.A.); francesca.spyrakis@unito.it (F.S.)
5 Laboratory Medicine Department, Laboratory of Molecular Virology, Maggiore della Carità Hospital, 28100 Novara, Italy; paolo.ravanini@gmail.com (P.R.)
6 Center for Translational Research on Autoimmune and Allergic Disease-CAAD, Novara, Italy.

* Correspondence: valentina.delloste@unito.it; Tel.: +39-011-6705631 (V.D.O.); marco.deandrea@unito.it; Tel.: +39-011-6705647 (M.D.A.)

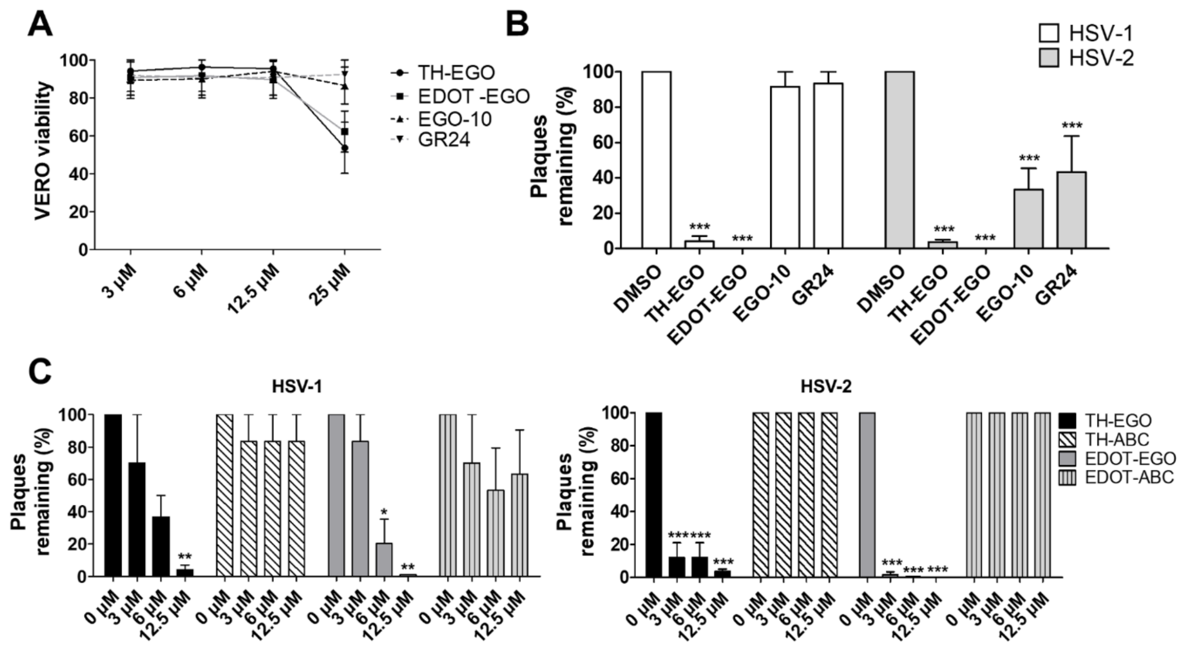

Figure S1. SL analogs antiviral activity against HSV. (**A**) Cell viability of VERO cells upon SL analogs treatment. Confluent VERO cells seeded in 96-well plates were incubated with different concentrations of the indicated SLs for 48 h and then processed for MTT assay. Graphs are representative of three independent experiments with duplicate replicate wells for each analysis. (**B**) Identification of SL-derivatives with anti-HSV-1 or –HSV-2 activity. VEROs were cultivated in 24-well plates, pre-treated with 12.5 μM of the indicated SLs for 2 h. Later, cells were infected with HSV-1 or HSV-2 (MOI of 0.1) and following virus adsorption (2 h at 37 °C), the viral inoculum was removed; cultures were exposed to SLs during the infection and until infected and DMSO threated control cultures displayed extensive cytopathology (48 h). DMSO was employed as vehicle control. The extent of HSV replication was then assessed by titrating the infectivity of supernatants and cell-associated viruses -obtained from freeze (liquid nitrogen)/thaw (37 °C) cycles- combined by a standard plaque assay on VEROs. Plaques were microscopically counted, and the mean plaque counts for each molecule are expressed as a percentage of the mean plaque count of infected and DMSO threated control cultures. Three independent experiments were performed, and one representative is shown (***, P<0.001, two-way ANOVA followed by Bonferroni's post-tests). (**C**) VEROs were infected with HSV-1 (left panel) or HSV-2 (right panel) (MOI of 0.1) and, where indicated, the cells were treated with increasing concentrations of the indicated SL derivatives, or DMSO, before as well as during virus adsorption. These remained in the culture media throughout the experiment. The extent of HSV replication was then assessed by titrating the infectivity of supernatants and cells combined by standard plaque assay. Plaques were microscopically counted, and the mean plaque counts for each drug concentration were expressed as a percentage of the mean count of infected and DMSO threated control cultures. The number of plaques was plotted as a function of drug concentration. Three independent experiments were performed, and one representative is shown (*, P<0.05; **, P<0.01; ***, P<0.001, two-way ANOVA followed by Bonferroni's post-tests).

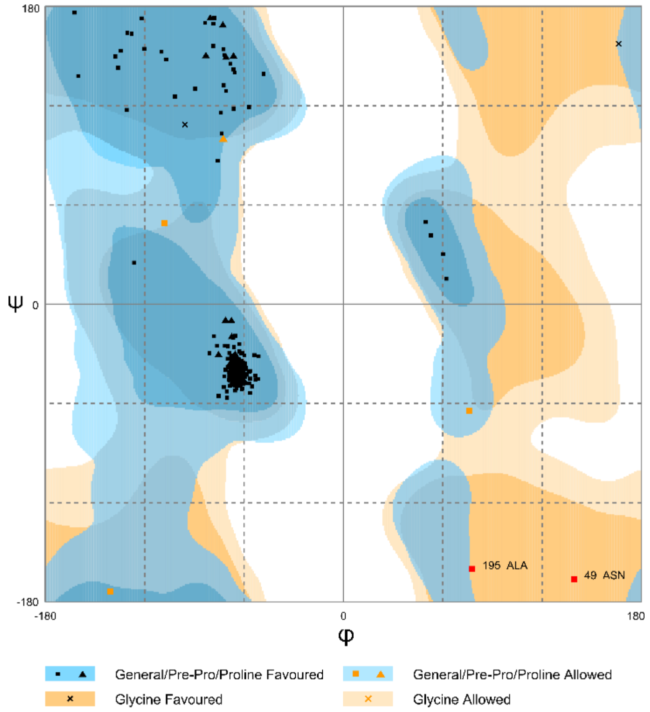

Figure S2. Ramachandran plot of IE1 homology model.

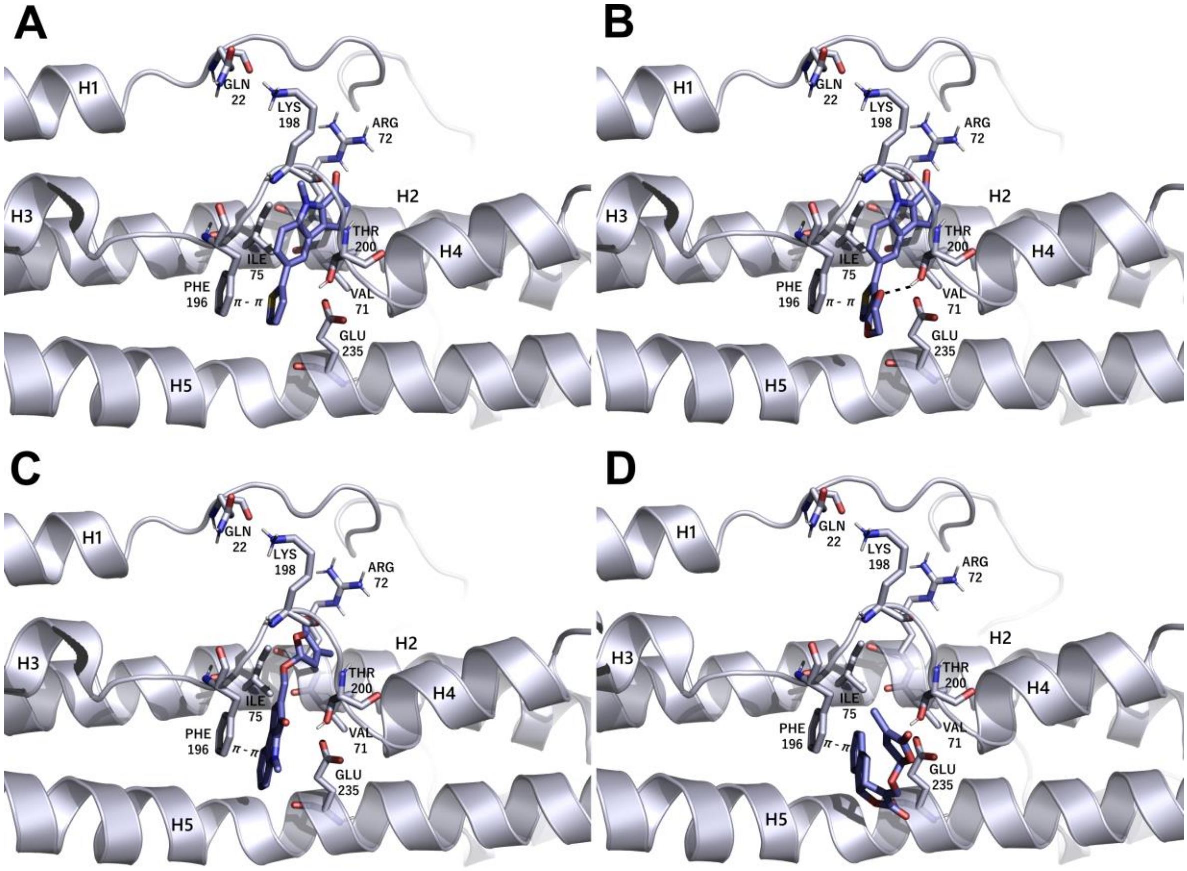

Figure S3. Predicted binding poses of TH-ABC (**A**), EDOT-ABC (**B**), EGO-10 (**C**) and GR24 (**D**) in IE1 protein model. Hydrogen bonds are shown as black dashed lines. The SLs and the residues lining the pocket are displayed as lilac and grey capped sticks, respectively.
